# Supplementary material for: Hot spot prediction in protein-protein interactions by an ensemble system
Source: BMC Syst Biol. 2018 Dec 31;12(Suppl 9):132. doi: 10.1186/s12918-018-0665-8 (PMC6311905; doi:10.1186/s12918-018-0665-8)
Supplement: Supplementary file 2 — All relevant MATLAB code. A simple Matlab implement of our predictor. (ZIP 19,742 kb) [file 12918_2018_665_MOESM2_ESM.zip › Additional file 2/Read ASA/ASA website/dbMPIKT/4O27B.html]

 NetSurfP - Protein Surface Accessibility and Secondary Structure Predictions

  

|  |  |  |
| --- | --- | --- |
|  |  | NetSurfP - Protein Surface Accessibility and Secondary Structure Predictions  Technical University of Denmark |

  


---

```
# For publication of results, please cite:
# A generic method for assignment of reliability scores applied to solvent accessibility predictions.
# Bent Petersen, Thomas Nordahl Petersen, Pernille Andersen, Morten Nielsen and Claus Lundegaard
# BMC Structural Biology 2009, 9:51 doi:10.1186/1472-6807-9-51
#
# Column 1: Class assignment - B for buried or E for Exposed - Threshold: 25% exposure, but not based on RSA
# Column 2: Amino acid
# Column 3: Sequence name
# Column 4: Amino acid number
# Column 5: Relative Surface Accessibility - RSA
# Column 6: Absolute Surface Accessibility
# Column 7: Z-fit score for RSA prediction
# Column 8: Probability for Alpha-Helix
# Column 9: Probability for Beta-strand
# Column 10: Probability for Coil
E M  4O27B                  1    0.706 141.231  -2.159   0.003   0.003   0.994
E A  4O27B                  2    0.533  58.737  -0.256   0.003   0.003   0.994
E D  4O27B                  3    0.475  68.491   0.251   0.016   0.005   0.979
E P  4O27B                  4    0.294  41.676  -0.303   0.502   0.002   0.495
E E  4O27B                  5    0.599 104.593   0.708   0.694   0.003   0.303
E E  4O27B                  6    0.608 106.305   1.060   0.717   0.014   0.269
E L  4O27B                  7    0.343  62.712   1.462   0.751   0.050   0.199
B F  4O27B                  8    0.075  15.093   0.696   0.701   0.107   0.192
E T  4O27B                  9    0.567  78.685   1.446   0.649   0.163   0.188
E K  4O27B                 10    0.378  77.734   1.334   0.649   0.163   0.188
B L  4O27B                 11    0.190  34.807   0.703   0.538   0.173   0.289
E E  4O27B                 12    0.472  82.511   0.878   0.307   0.165   0.527
E K  4O27B                 13    0.306  62.924   1.343   0.352   0.332   0.316
B I  4O27B                 14    0.061  11.377   0.490   0.135   0.317   0.548
B G  4O27B                 15    0.183  14.418  -0.993   0.064   0.216   0.721
E K  4O27B                 16    0.535 109.988   0.387   0.019   0.141   0.840
B G  4O27B                 17    0.239  18.825  -2.329   0.018   0.088   0.893
B S  4O27B                 18    0.209  24.553  -1.645   0.018   0.088   0.893
E F  4O27B                 19    0.313  62.839  -1.028   0.021   0.279   0.699
B G  4O27B                 20    0.072   5.666  -0.917   0.022   0.552   0.426
B E  4O27B                 21    0.212  37.106   1.344   0.018   0.846   0.136
B V  4O27B                 22    0.026   3.919   0.716   0.006   0.962   0.032
B F  4O27B                 23    0.120  24.004   1.258   0.001   0.959   0.040
B K  4O27B                 24    0.122  25.157   1.322   0.000   0.983   0.017
B G  4O27B                 25    0.017   1.338   0.497   0.000   0.983   0.017
B I  4O27B                 26    0.192  35.464   1.390   0.000   0.983   0.017
B D  4O27B                 27    0.184  26.572   0.346   0.001   0.959   0.040
B N  4O27B                 28    0.250  36.600   0.855   0.004   0.514   0.481
E R  4O27B                 29    0.689 157.689  -0.113   0.004   0.197   0.799
E T  4O27B                 30    0.588  81.597   0.030   0.005   0.015   0.979
E Q  4O27B                 31    0.539  96.283  -0.004   0.005   0.015   0.979
E K  4O27B                 32    0.331  68.169   1.457   0.004   0.514   0.481
B V  4O27B                 33    0.202  31.063   1.954   0.001   0.959   0.040
B V  4O27B                 34    0.053   8.208   1.951   0.001   0.959   0.040
B A  4O27B                 35    0.019   2.116   0.972   0.001   0.959   0.040
B I  4O27B                 36    0.050   9.158   0.871   0.000   0.983   0.017
B K  4O27B                 37    0.079  16.333   0.602   0.000   0.983   0.017
B I  4O27B                 38    0.187  34.503   1.106   0.001   0.959   0.040
B I  4O27B                 39    0.063  11.581   0.978   0.001   0.959   0.040
E D  4O27B                 40    0.474  68.275   1.555   0.002   0.816   0.182
B L  4O27B                 41    0.107  19.537   0.198   0.005   0.262   0.733
E E  4O27B                 42    0.726 126.850   0.814   0.005   0.045   0.951
E E  4O27B                 43    0.641 112.000   0.687   0.005   0.015   0.979
E A  4O27B                 44    0.451  49.667   0.352   0.016   0.005   0.979
E E  4O27B                 45    0.684 119.407   0.672   0.858   0.002   0.139
E D  4O27B                 46    0.687  98.939   1.426   0.923   0.002   0.076
E E  4O27B                 47    0.305  53.371   1.154   0.923   0.002   0.076
B I  4O27B                 48    0.189  34.947   1.088   0.970   0.001   0.030
E E  4O27B                 49    0.577 100.854   1.393   0.970   0.001   0.030
E D  4O27B                 50    0.404  58.260   2.086   0.970   0.001   0.030
B I  4O27B                 51    0.020   3.774   1.086   0.988   0.000   0.012
E Q  4O27B                 52    0.297  53.044   1.257   0.988   0.000   0.012
E Q  4O27B                 53    0.436  77.905   1.495   0.970   0.001   0.030
B E  4O27B                 54    0.061  10.587   0.051   0.988   0.000   0.012
B I  4O27B                 55    0.046   8.547   0.552   0.988   0.000   0.012
E T  4O27B                 56    0.456  63.233   1.694   0.988   0.000   0.012
B V  4O27B                 57    0.108  16.646   0.550   0.970   0.001   0.030
B L  4O27B                 58    0.079  14.502   0.073   0.970   0.001   0.030
E S  4O27B                 59    0.433  50.794   0.573   0.858   0.002   0.139
E Q  4O27B                 60    0.545  97.248   0.985   0.600   0.003   0.397
B C  4O27B                 61    0.079  11.021   0.828   0.176   0.004   0.820
E D  4O27B                 62    0.674  97.138   0.390   0.016   0.005   0.979
B S  4O27B                 63    0.157  18.354   0.160   0.003   0.003   0.994
E P  4O27B                 64    0.606  86.048   1.040   0.018   0.019   0.964
B Y  4O27B                 65    0.215  45.924  -0.244   0.052   0.084   0.864
B V  4O27B                 66    0.040   6.179   0.792   0.021   0.451   0.528
B T  4O27B                 67    0.057   7.892   0.687   0.021   0.756   0.223
E K  4O27B                 68    0.315  64.754   1.067   0.018   0.846   0.136
B Y  4O27B                 69    0.050  10.642   0.690   0.018   0.846   0.136
B Y  4O27B                 70    0.183  39.214   1.046   0.021   0.756   0.223
B G  4O27B                 71    0.110   8.626  -2.010   0.002   0.816   0.182
B S  4O27B                 72    0.076   8.942  -0.051   0.001   0.900   0.099
B Y  4O27B                 73    0.141  30.068   0.150   0.001   0.959   0.040
E L  4O27B                 74    0.328  60.057   0.655   0.001   0.959   0.040
E K  4O27B                 75    0.446  91.722   0.680   0.003   0.718   0.279
E D  4O27B                 76    0.709 102.138   0.343   0.005   0.045   0.951
E T  4O27B                 77    0.546  75.702  -0.104   0.005   0.015   0.979
B K  4O27B                 78    0.232  47.640   1.770   0.003   0.718   0.279
B L  4O27B                 79    0.023   4.285   1.129   0.001   0.959   0.040
B W  4O27B                 80    0.064  15.272   1.287   0.006   0.962   0.032
B I  4O27B                 81    0.020   3.774   1.601   0.001   0.959   0.040
B I  4O27B                 82    0.020   3.626   1.388   0.001   0.959   0.040
B M  4O27B                 83    0.023   4.702   1.398   0.011   0.918   0.071
B E  4O27B                 84    0.055   9.609   0.358   0.002   0.816   0.182
B Y  4O27B                 85    0.089  18.934   0.407   0.004   0.616   0.381
B L  4O27B                 86    0.038   6.921   0.011   0.019   0.141   0.840
E G  4O27B                 87    0.494  38.846  -0.181   0.005   0.015   0.979
E G  4O27B                 88    0.359  28.269  -1.577   0.058   0.017   0.925
B G  4O27B                 89    0.194  15.299  -1.043   0.115   0.016   0.868
B S  4O27B                 90    0.197  23.112  -0.188   0.339   0.016   0.645
B A  4O27B                 91    0.037   4.088  -0.351   0.923   0.002   0.076
E L  4O27B                 92    0.306  56.102   0.953   0.923   0.002   0.076
E D  4O27B                 93    0.429  61.833   1.160   0.923   0.002   0.076
B L  4O27B                 94    0.119  21.844   0.780   0.923   0.002   0.076
B L  4O27B                 95    0.078  14.300   0.197   0.858   0.002   0.139
E E  4O27B                 96    0.547  95.613   1.291   0.600   0.003   0.397
E P  4O27B                 97    0.685  97.258   0.651   0.176   0.004   0.820
E G  4O27B                 98    0.482  37.933   0.106   0.016   0.005   0.979
E P  4O27B                 99    0.567  80.486   0.335   0.005   0.015   0.979
B L  4O27B                100    0.055   9.997  -0.033   0.005   0.015   0.979
E D  4O27B                101    0.531  76.459   1.330   0.016   0.005   0.979
B E  4O27B                102    0.121  21.174  -0.316   0.923   0.002   0.076
E T  4O27B                103    0.591  81.972   0.751   0.970   0.001   0.030
B Q  4O27B                104    0.159  28.433   1.610   0.970   0.001   0.030
B I  4O27B                105    0.015   2.868   1.334   0.970   0.001   0.030
B A  4O27B                106    0.076   8.342   0.939   0.970   0.001   0.030
E T  4O27B                107    0.317  44.023   1.823   0.970   0.001   0.030
B I  4O27B                108    0.053   9.842   1.455   0.970   0.001   0.030
B L  4O27B                109    0.018   3.296   1.346   0.988   0.000   0.012
B R  4O27B                110    0.205  46.945   1.438   0.988   0.000   0.012
B E  4O27B                111    0.149  26.083   0.516   0.988   0.000   0.012
B I  4O27B                112    0.027   5.069   1.149   0.988   0.000   0.012
B L  4O27B                113    0.035   6.409  -0.085   0.988   0.000   0.012
E K  4O27B                114    0.287  59.118   1.224   0.970   0.001   0.030
B G  4O27B                115    0.034   2.715  -0.921   0.970   0.001   0.030
B L  4O27B                116    0.037   6.756   0.457   0.970   0.001   0.030
E D  4O27B                117    0.354  50.968   1.234   0.970   0.001   0.030
B Y  4O27B                118    0.211  45.027   0.748   0.970   0.001   0.030
B L  4O27B                119    0.039   7.196   0.510   0.923   0.002   0.076
B H  4O27B                120    0.220  40.036   0.856   0.782   0.003   0.216
E S  4O27B                121    0.748  87.619   1.131   0.406   0.004   0.590
E E  4O27B                122    0.551  96.277   1.265   0.016   0.005   0.979
E K  4O27B                123    0.539 110.893   0.086   0.005   0.015   0.979
B K  4O27B                124    0.168  34.599   1.256   0.004   0.197   0.799
B I  4O27B                125    0.113  20.849   0.351   0.022   0.552   0.426
B H  4O27B                126    0.158  28.795   0.549   0.021   0.451   0.528
B R  4O27B                127    0.246  56.380   0.381   0.066   0.296   0.638
B D  4O27B                128    0.204  29.425  -0.473   0.056   0.142   0.802
B I  4O27B                129    0.075  13.893   1.165   0.052   0.084   0.864
E K  4O27B                130    0.322  66.338   0.934   0.053   0.043   0.903
B A  4O27B                131    0.122  13.389  -0.718   0.053   0.043   0.903
E A  4O27B                132    0.403  44.367   0.098   0.018   0.047   0.935
B N  4O27B                133    0.117  17.100  -0.569   0.064   0.216   0.721
B V  4O27B                134    0.059   9.145   0.578   0.023   0.655   0.322
B L  4O27B                135    0.088  16.058   0.525   0.021   0.756   0.223
B L  4O27B                136    0.085  15.600   0.868   0.023   0.655   0.322
B S  4O27B                137    0.136  15.927  -0.077   0.020   0.205   0.775
E E  4O27B                138    0.615 107.388   0.220   0.018   0.047   0.935
E H  4O27B                139    0.707 128.512   0.770   0.005   0.015   0.979
B G  4O27B                140    0.184  14.457  -0.137   0.018   0.019   0.964
E E  4O27B                141    0.394  68.744   1.379   0.021   0.279   0.699
B V  4O27B                142    0.038   5.764   0.380   0.018   0.846   0.136
B K  4O27B                143    0.148  30.444   1.425   0.011   0.918   0.071
B L  4O27B                144    0.040   7.287   0.315   0.011   0.918   0.071
B A  4O27B                145    0.038   4.155  -0.216   0.018   0.846   0.136
B D  4O27B                146    0.080  11.542  -1.168   0.079   0.592   0.329
B F  4O27B                147    0.081  16.237  -0.152   0.216   0.235   0.548
B G  4O27B                148    0.097   7.642  -1.435   0.453   0.248   0.299
B V  4O27B                149    0.093  14.309   0.406   0.594   0.285   0.121
B A  4O27B                150    0.034   3.747  -0.818   0.662   0.225   0.113
B G  4O27B                151    0.229  18.007   0.422   0.662   0.225   0.113
E Q  4O27B                152    0.313  55.848   0.704   0.594   0.285   0.121
B L  4O27B                153    0.074  13.568   0.085   0.453   0.248   0.299
E T  4O27B                154    0.387  53.649   0.385   0.502   0.102   0.396
E D  4O27B                155    0.619  89.227   0.034   0.354   0.048   0.598
E T  4O27B                156    0.524  72.651  -0.624   0.354   0.048   0.598
E Q  4O27B                157    0.549  98.105  -1.442   0.339   0.016   0.645
E I  4O27B                158    0.503  93.037  -0.429   0.181   0.016   0.803
E K  4O27B                159    0.422  86.744  -0.075   0.113   0.043   0.844
B R  4O27B                160    0.254  58.097  -0.098   0.052   0.084   0.864
E N  4O27B                161    0.390  57.125   0.434   0.019   0.141   0.840
B E  4O27B                162    0.066  11.548  -0.240   0.021   0.451   0.528
B F  4O27B                163    0.082  16.377  -0.683   0.023   0.655   0.322
B V  4O27B                164    0.079  12.096  -0.595   0.021   0.756   0.223
B G  4O27B                165    0.046   3.644  -1.397   0.022   0.552   0.426
B T  4O27B                166    0.045   6.200   0.553   0.020   0.205   0.775
B P  4O27B                167    0.070   9.919  -0.114   0.019   0.141   0.840
B F  4O27B                168    0.094  18.906  -0.359   0.056   0.142   0.802
B W  4O27B                169    0.064  15.440  -0.329   0.066   0.296   0.638
B M  4O27B                170    0.042   8.344  -0.542   0.135   0.317   0.548
B A  4O27B                171    0.042   4.595   0.309   0.191   0.086   0.723
B P  4O27B                172    0.057   8.131  -0.016   0.386   0.097   0.517
B E  4O27B                173    0.090  15.688  -0.211   0.478   0.309   0.214
B V  4O27B                174    0.164  25.238   0.193   0.373   0.401   0.226
B I  4O27B                175    0.079  14.652   0.487   0.391   0.466   0.143
E K  4O27B                176    0.540 111.140   0.945   0.216   0.235   0.548
E Q  4O27B                177    0.743 132.753  -0.674   0.053   0.043   0.903
E S  4O27B                178    0.597  69.980   0.531   0.018   0.047   0.935
E A  4O27B                179    0.602  66.373  -1.182   0.018   0.047   0.935
B Y  4O27B                180    0.194  41.436  -0.221   0.018   0.047   0.935
E D  4O27B                181    0.542  78.045  -0.261   0.018   0.019   0.964
E S  4O27B                182    0.350  41.032   0.192   0.053   0.043   0.903
E K  4O27B                183    0.343  70.514   0.716   0.191   0.086   0.723
B A  4O27B                184    0.030   3.350   0.663   0.278   0.093   0.628
B D  4O27B                185    0.055   7.868   0.615   0.605   0.105   0.290
B I  4O27B                186    0.026   4.847   0.953   0.779   0.100   0.120
B W  4O27B                187    0.048  11.448   0.007   0.831   0.044   0.125
B S  4O27B                188    0.033   3.914  -0.949   0.938   0.007   0.055
B L  4O27B                189    0.041   7.562   0.264   0.938   0.007   0.055
B G  4O27B                190    0.039   3.093  -0.643   0.938   0.007   0.055
B I  4O27B                191    0.026   4.736   0.818   0.938   0.007   0.055
B T  4O27B                192    0.040   5.603   0.576   0.975   0.003   0.022
B A  4O27B                193    0.020   2.182   1.256   0.970   0.001   0.030
B I  4O27B                194    0.046   8.473   1.074   0.988   0.000   0.012
B E  4O27B                195    0.034   5.905   0.082   0.970   0.001   0.030
B L  4O27B                196    0.035   6.335   0.372   0.970   0.001   0.030
B A  4O27B                197    0.038   4.166   0.175   0.858   0.002   0.139
B R  4O27B                198    0.189  43.258  -0.199   0.502   0.002   0.495
E G  4O27B                199    0.263  20.714  -0.894   0.109   0.005   0.886
E E  4O27B                200    0.346  60.499   0.514   0.016   0.005   0.979
B P  4O27B                201    0.231  32.736   0.568   0.016   0.005   0.979
B P  4O27B                202    0.101  14.360  -1.554   0.003   0.003   0.994
B H  4O27B                203    0.108  19.663  -0.188   0.003   0.003   0.994
E S  4O27B                204    0.432  50.666  -0.135   0.003   0.003   0.994
E E  4O27B                205    0.747 130.483  -0.658   0.003   0.003   0.994
E L  4O27B                206    0.311  56.962  -0.194   0.053   0.005   0.942
E H  4O27B                207    0.485  88.203  -0.082   0.016   0.005   0.979
B P  4O27B                208    0.158  22.378  -0.489   0.923   0.002   0.076
B M  4O27B                209    0.241  48.224  -0.165   0.970   0.001   0.030
E K  4O27B                210    0.368  75.615   1.435   0.970   0.001   0.030
B V  4O27B                211    0.029   4.457   0.059   0.970   0.001   0.030
B L  4O27B                212    0.049   8.899   0.401   0.970   0.001   0.030
B F  4O27B                213    0.253  50.797   0.697   0.975   0.003   0.022
B L  4O27B                214    0.235  43.047   1.331   0.970   0.001   0.030
B I  4O27B                215    0.035   6.456   0.140   0.858   0.002   0.139
B P  4O27B                216    0.241  34.184   0.017   0.694   0.003   0.303
E K  4O27B                217    0.654 134.569   0.119   0.246   0.004   0.750
E N  4O27B                218    0.644  94.326  -0.630   0.053   0.005   0.942
E N  4O27B                219    0.695 101.704  -0.130   0.003   0.003   0.994
E P  4O27B                220    0.310  43.989  -0.287   0.003   0.003   0.994
E P  4O27B                221    0.295  41.889  -0.386   0.003   0.003   0.994
E T  4O27B                222    0.642  89.115   0.716   0.003   0.003   0.994
B L  4O27B                223    0.082  14.959  -0.113   0.003   0.003   0.994
E E  4O27B                224    0.343  60.009   0.587   0.053   0.005   0.942
E G  4O27B                225    0.844  66.439   0.006   0.321   0.003   0.675
E N  4O27B                226    0.572  83.697   1.160   0.406   0.004   0.590
B Y  4O27B                227    0.073  15.643   0.782   0.321   0.003   0.675
B S  4O27B                228    0.107  12.564   0.001   0.181   0.016   0.803
E K  4O27B                229    0.713 146.705   1.124   0.923   0.002   0.076
E P  4O27B                230    0.373  52.872   1.335   0.970   0.001   0.030
B L  4O27B                231    0.019   3.387   1.117   0.970   0.001   0.030
E K  4O27B                232    0.289  59.406   1.690   0.970   0.001   0.030
E E  4O27B                233    0.481  84.101   1.539   0.970   0.001   0.030
B F  4O27B                234    0.047   9.433   0.974   0.970   0.001   0.030
B V  4O27B                235    0.023   3.458   1.154   0.988   0.000   0.012
E E  4O27B                236    0.346  60.411   1.954   0.970   0.001   0.030
E A  4O27B                237    0.341  37.534   2.120   0.970   0.001   0.030
B C  4O27B                238    0.025   3.454   0.847   0.923   0.002   0.076
B L  4O27B                239    0.047   8.697   0.993   0.858   0.002   0.139
E N  4O27B                240    0.480  70.287   1.603   0.600   0.003   0.397
E K  4O27B                241    0.392  80.717   1.427   0.321   0.003   0.675
B E  4O27B                242    0.226  39.517   0.681   0.016   0.005   0.979
B P  4O27B                243    0.149  21.115  -0.299   0.016   0.005   0.979
E S  4O27B                244    0.683  79.989  -0.103   0.016   0.005   0.979
E F  4O27B                245    0.476  95.433  -0.084   0.016   0.005   0.979
B R  4O27B                246    0.208  47.632  -0.447   0.018   0.019   0.964
B P  4O27B                247    0.255  36.199  -0.718   0.058   0.017   0.925
E T  4O27B                248    0.268  37.199   0.711   0.058   0.017   0.925
B A  4O27B                249    0.035   3.901  -0.546   0.923   0.002   0.076
E K  4O27B                250    0.454  93.450   1.361   0.923   0.002   0.076
E E  4O27B                251    0.298  52.008   1.003   0.923   0.002   0.076
B L  4O27B                252    0.037   6.775  -0.190   0.858   0.002   0.139
B L  4O27B                253    0.074  13.568   0.247   0.782   0.003   0.216
E K  4O27B                254    0.636 130.866   0.755   0.502   0.002   0.495
B H  4O27B                255    0.172  31.323   1.440   0.246   0.004   0.750
E K  4O27B                256    0.434  89.315   0.327   0.522   0.016   0.462
B F  4O27B                257    0.140  27.998   0.321   0.660   0.049   0.291
B I  4O27B                258    0.055  10.175  -0.075   0.660   0.049   0.291
E L  4O27B                259    0.347  63.536   1.221   0.751   0.050   0.199
E R  4O27B                260    0.607 138.957   0.924   0.622   0.015   0.363
E N  4O27B                261    0.367  53.670   0.653   0.430   0.016   0.555
E A  4O27B                262    0.337  37.115  -0.501   0.181   0.016   0.803
E K  4O27B                263    0.676 138.991   0.216   0.115   0.016   0.868
E K  4O27B                264    0.533 109.576  -0.045   0.115   0.016   0.868
E T  4O27B                265    0.423  58.656  -0.346   0.257   0.016   0.727
E S  4O27B                266    0.563  66.019  -0.523   0.522   0.016   0.462
E Y  4O27B                267    0.366  78.257   0.948   0.502   0.002   0.495
B L  4O27B                268    0.040   7.306  -0.185   0.858   0.002   0.139
E T  4O27B                269    0.399  55.286   1.925   0.923   0.002   0.076
E E  4O27B                270    0.564  98.513   1.785   0.970   0.001   0.030
B L  4O27B                271    0.102  18.731   1.365   0.970   0.001   0.030
B I  4O27B                272    0.053   9.731   0.802   0.970   0.001   0.030
E D  4O27B                273    0.567  81.734   1.880   0.970   0.001   0.030
E R  4O27B                274    0.383  87.684   1.623   0.970   0.001   0.030
B Y  4O27B                275    0.075  16.028   1.024   0.970   0.001   0.030
E K  4O27B                276    0.415  85.386   1.000   0.923   0.002   0.076
E R  4O27B                277    0.659 151.003   1.392   0.858   0.002   0.139
B W  4O27B                278    0.207  49.832   0.375   0.694   0.003   0.303
E K  4O27B                279    0.567 116.611   1.002   0.406   0.004   0.590
E A  4O27B                280    0.793  87.345   0.401   0.109   0.005   0.886
E E  4O27B                281    0.869 151.744   1.182   0.003   0.003   0.994

Explain the output.  Go back.
```

---
